# Supplementary material for: MoS2 nanosheet incorporated α-Fe2O3/ZnO nanocomposite with enhanced photocatalytic dye degradation and hydrogen production ability
Source: RSC Adv. 2019 Dec 5;9(69):40357–67. doi: 10.1039/c9ra07526g (PMC9076241; doi:10.1039/c9ra07526g)
Supplement: RA-009-C9RA07526G-s001 [file RA-009-C9RA07526G-s001.pdf]

Electronic Supplementary Material (ESI) for RSC Advances.
This journal is © The Royal Society of Chemistry 2019

Electronic Supplementary Information

MoS_2 nanosheets incorporated α-Fe_2O_3/ZnO nanocomposite with enhanced photocatalytic dye degradation and hydrogen production ability

Angkita Mistry Tama[a†], Subrata Das[a†], Sagar Dutta[a], M D I Bhuyan[a], M. N. Islam[b] and M. A. Basith[a]

[a] Nanotechnology Research Laboratory, Department of Physics, Bangladesh University of Engineering and Technology, Dhaka-1000, Bangladesh.

[b] Department of Chemistry, Bangladesh University of Engineering and Technology, Dhaka-1000, Bangladesh.

†These authors contributed equally.

Table S1 Atomic coordinates, constituent crystallographic phases (in wt%) of synthesized nanomaterials and reliability (R) factors obtained from Rietveld refined powder spectra. Numbers in the parentheses indicate errors in the last significant digit.

Sample	Atomic Coordinates	Crystallographic Phase (in wt%)	R Factor
α-Fe_2O_3	Fe (0,0, 0.3557)<br>O (0.3176,0,0.25)	Trigonal (100%)	$R_{wp} = 2.83$<br>$R_p = 2.25$<br>$R_{Exp} = 2.90$<br>$\chi^2 = 0.953$
α-Fe_2O_3/ZnO	Fe (0,0, 0.3556)<br>O (0.3052,0,0.25)<br>Zn (0.33333,0.66667,0)<br>O (0.33333,0.66667, 0.3919)	Trigonal (80%)<br>Hexagonal (20%)	$R_{wp} = 3.01$<br>$R_p = 2.41$<br>$R_{Exp} = 2.94$<br>$\chi^2 = 1.00$
NMS inc. α-Fe_2O_3/ZnO	Fe (0,0, 0.3563)<br>O (0.3067,0,0.25)<br>Zn (0.33333,0.66667,0)<br>O (0.33333,0.66667, 0.3663)<br>Mo (0.33333,0.66667,0)<br>S (0.33333,0.66667, 0.6243)	Trigonal (17%)<br>Hexagonal (83%)	$R_{wp} = 4.34$<br>$R_p = 3.27$<br>$R_{Exp} = 3.27$<br>$\chi^2 = 1.77$
UMS inc. α-Fe_2O_3/ZnO	Fe (0,0, 0.3547)<br>O (0.3121,0,0.25)<br>Zn (0.33333,0.66667,0)<br>O (0.33333,0.66667, 0.3508)<br>Mo (0.33333,0.66667,0)<br>S (0.33333,0.66667, 0.6243)	Trigonal (24%)<br>Hexagonal (76%)	$R_{wp} = 2.73$<br>$R_p = 2.19$<br>$R_{Exp} = 2.67$<br>$\chi^2 = 1.05$

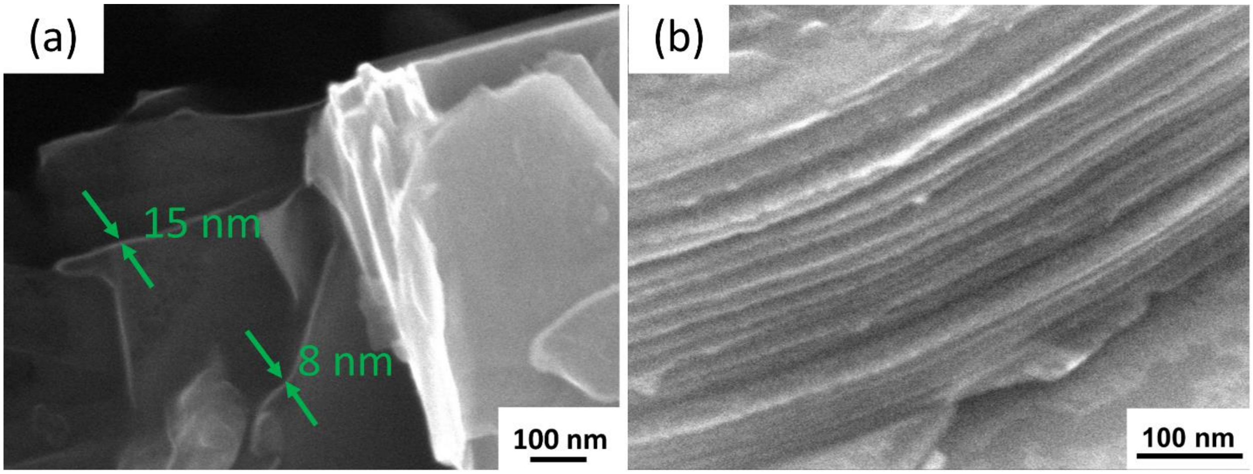

Fig. S1 FESEM images of ultrasonicated few-layer MoS_2 nanosheets with two different magnifications. The layer thickness of two nanosheets is marked in (a) and an overall view of successfully delaminated few-layer nanosheets of MoS_2 is demonstrated in (b).

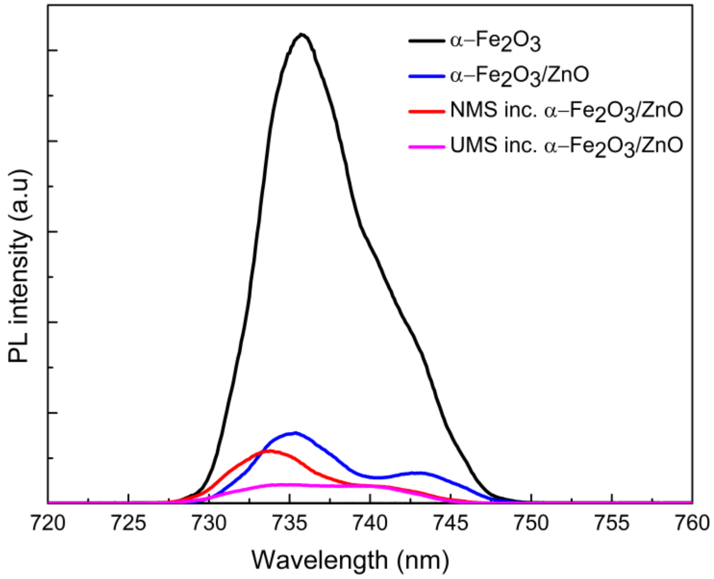

Fig. S2 (a) Steady-state photoluminescence intensity spectra of synthesized α-Fe_2O_3 nanoparticles, α-Fe_2O_3 /ZnO, non-ultrasonicated MoS_2 incorporated α-Fe_2O_3 /ZnO and ultrasonicated MoS_2 incorporated α-Fe_2O_3 /ZnO nanocomposites.

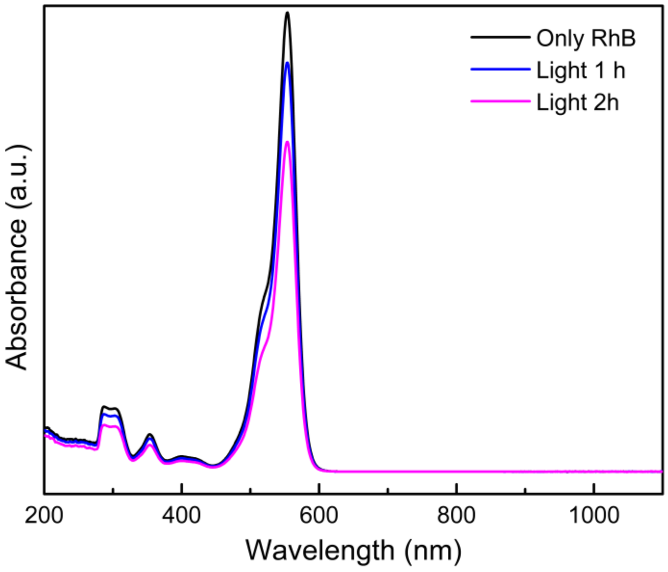

Fig. S3 Absorption spectra of RhB dye solution before and after being photodegraded by ultrasonicated MoS_2 incorporated α-Fe_2O_3/ZnO under UV-visible light irradiation.

Table S2 Amount of produced hydrogen gas by as-synthesized ultrasonicated MoS_2 incorporated α-Fe_2O_3/ZnO and other analogous nanocomposites

Photocatalyst	Light Source	Amount of produced hydrogen gas after 4 hours of irradiation (μmol H_2 /g catalyst)	Reference
UMS inc. α-Fe_2O_3/ZnO	500 W Hg-Xe lamp	~614	Present work
MoS_2/RGO	300 W Xe lamp	~190	[1]
WO_3/g-C_3N_4	Artificial solar light	~250	[2]
TiO_2/$MoSe_2$	300 W Xe lamp	~20	[3]

Calculation of Conduction Band Minima (CBM) and Valence Band Maxima (VBM)

The Fermi energy (E_F) of a semiconductor material can be calculated theoretically using the empirical formula[4]:

$$E_F = \chi - E_c \qquad (1)$$

Here, χ is the absolute electronegativity and E_c is the free electron energy on hydrogen scale (approximately 4.5 eV). For compound semiconductors, χ is calculated using the geometric mean of constituent atoms' absolute electronegativity.[5] Usually for semiconductor, Fermi energy denotes the

midlevel of the gap between CBM and VBM. Hence, the CBM and VBM of a semiconductor can be calculated using the following equations:

$$CBM = E_F - 0.5E_g \qquad (2)$$

$$VBM = E_F + 0.5E_g \qquad (3)$$

Here, E_g denotes the bandgap energy.

The calculated values of χ, E_F, CBM and VBM for α-Fe_2O_3, ZnO and MoS_2 nanosheets are presented in Table S3. For α-Fe_2O_3, the experimentally obtained bandgap (E_g) value is used whereas for ZnO and few-layer MoS_2 nanosheets, we have considered the bandgap values as reported by previous investigations.[6,7]

Table S3 Obtained values of absolute electronegativity (χ), bandgap energy (E_g), Fermi energy (E_F), CBM and VBM of α-Fe_2O_3, ZnO and few-layer MoS_2 nanosheets.

**Material**	**χ (eV)**	**E_g(eV)**	**E_F (eV)**	**CBM (eV)**	**VBM (eV)**
α-Fe_2O_3	5.88618	1.87	1.386	0.451	2.321
ZnO	5.79250	3.37	1.293	-0.393	2.978
MoS_2 Nanosheets	5.32372	1.25	0.824	0.199	1.449

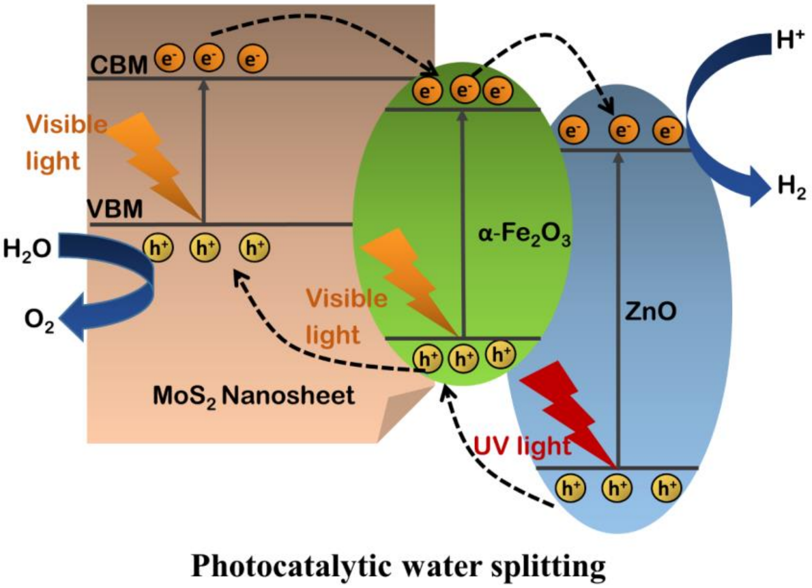

Fig. S4 Schematic of proposed photocatalytic water splitting mechanism using ultrasonicated MoS_2 incorporated α-Fe_2O_3/ZnO nanocomposite as catalyst under solar light irradiation

References

1. S. Min and G. Lu, *J. Phys. Chem. C*, 2012, **116,** 25415-25424.
2. H. Katsumata, Y. Tachi, T. Suzuki and S. Kaneco, *RSC Adv*., 2014, **4**, 21405-21409.
3. L. Wu, S. Shi, Q. Li, X. Zhang and X. Cui, *Int. J. Hydrog. Energy*, 2019, **44**, 720-728.
4. Y. Cui, S. M. Goldup and S. Dunn, *RSC Adv.*, 2015, **5**, 30372-30379.
5. R. G. Pearson, *Inorganic chem.*, 1988, **27**, 734-740.
6. S. Das, A. M. Tama, S. Dutta, M. S. Ali and M. A. Basith, *Mater. Res. Express*, 2019. DOI:10.1088/2053-1591/ab57dd
7. V. Srikant and D. Clarke, *J. Appl. Phys.*, 1998, **83**, 5447-5451.
